# Conventionally used reference genes are not outstanding for normalization of gene expression in human cancer research

**DOI:** 10.1186/s12859-019-2809-2

**Published:** 2019-05-29

**Authors:** Jihoon Jo, Sunkyung Choi, Jooseong Oh, Sung-Gwon Lee, Song Yi Choi, Kee K. Kim, Chungoo Park

**Affiliations:** 1 School of Biological Sciences and Technology, Chonnam National University, 77 Yongbong-Ro, Buk-Ku, GwangJu, 61186 Republic of Korea; 2 Department of Biochemistry, Chungnam National University, 99 Daehak-Ro, Yuseong-Ku, Daejeon, 34134 Republic of Korea; 3 Department of Pathology, Chungnam National University, 282 Munhwa-Ro, Jung-Ku, Daejeon, 35015 Republic of Korea

**Keywords:** RT-qPCR, Reference genes, Human cancer

## Abstract

**Background:**

The selection of reference genes is essential for quantifying gene expression. Theoretically they should be expressed stably and not regulated by experimental or pathological conditions. However, identification and validation of reference genes for human cancer research are still being regarded as a critical point, because cancerous tissues often represent genetic instability and heterogeneity. Recent pan-cancer studies have demonstrated the importance of the appropriate selection of reference genes for use as internal controls for the normalization of gene expression; however, no stably expressed, consensus reference genes valid for a range of different human cancers have yet been identified.

**Results:**

In the present study, we used large-scale cancer gene expression datasets from The Cancer Genome Atlas (TCGA) database, which contains 10,028 (9,364 cancerous and 664 normal) samples from 32 different cancer types, to confirm that the expression of the most commonly used reference genes is not consistent across a range of cancer types. Furthermore, we identified 38 novel candidate reference genes for the normalization of gene expression, independent of cancer type. These genes were found to be highly expressed and highly connected to relevant gene networks, and to be enriched in transcription-translation regulation processes. The expression stability of the newly identified reference genes across 29 cancerous and matched normal tissues were validated via quantitative reverse transcription PCR (RT-qPCR).

**Conclusions:**

We reveal that most commonly used reference genes in current cancer studies cannot be appropriate to serve as representative control genes for quantifying cancer-related gene expression levels, and propose in this study three potential reference genes (*HNRNPL*, *PCBP1*, and *RER1*) to be the most stably expressed across various cancerous and normal human tissues.

**Electronic supplementary material:**

The online version of this article (10.1186/s12859-019-2809-2) contains supplementary material, which is available to authorized users.

## Background

To understand how genetic alterations driving tumorigenesis lead to the formation of complex cellular networks and induce biological process variation, recent research into cancer genetics has focused on the identification of molecular differences between cancerous and normal tissues [1, 2]. Recent high-throughput transcriptomic studies [3] have offered the opportunity to explore the molecular complexity of human cancer, and have provided evidence for classifying human cancer data into normal, benign, and malignant classes, based on their gene expression patterns. Nevertheless, the expression levels of transcriptionally identified candidate cancer genes require experimental verification via molecular methods such as quantitative reverse transcription PCR (RT-qPCR). One of the most important factors ensuring the accuracy of RT-qPCR analyses is the normalization of the identified target-gene expression level to that of a consistently expressed reference gene. To date, cancer researchers have predominantly used the *GAPDH* and *β-actin* reference genes as internal reference controls, because their mRNA expression levels are established to be high and constant in many different cells and tissues [4, 5]. However, cancerous tissues often exhibit a higher level of gene expression variability than normal tissues, due to tumor heterogeneity, genetic instability, and the fact that genetic alterations in diverse cancer types may differentially affect cellular processes at the transcriptome level. Thus, it is a challenging to determine which reference genes would best serve as internal reference controls for a range of different human cancers. Indeed, an increasing number of researches have shown the striking expression variability of known reference genes in human cancers, and subsequently recommended novel reference genes for gene expression studies in each specific human cancer type [6, 7]. These efforts with in silico analysis (e.g., geNorm, NormFinder, and Bestkeeper [8–10]) are ongoing; however, to date, no transcriptome-wide analysis for the identification of the most stably expressed consensus reference genes has been reported.

The primary objective of the present study was to conduct a screen for the most stable reference genes for the study of cancer gene expression. We exploited large-scale gene expression data from The Cancer Genome Atlas (TCGA) database, which contains 10,028 (9,364 cancerous and 664 normal) samples from 32 different cancer types. We identified novel reference genes that exhibited both a high expression and low expression-variation level across various cancerous and normal tissue types, and then demonstrated the effectiveness of these newly identified reference genes for use in RT-qPCR. Thus, the results of the present study promote a better understanding of gene expression changes in different cancer types, and will be of considerable use in facilitating the normalization of target-gene expression levels in future cancer research.

## Methods

### Data collection and bioinformatics analysis

**Fig. 1:**
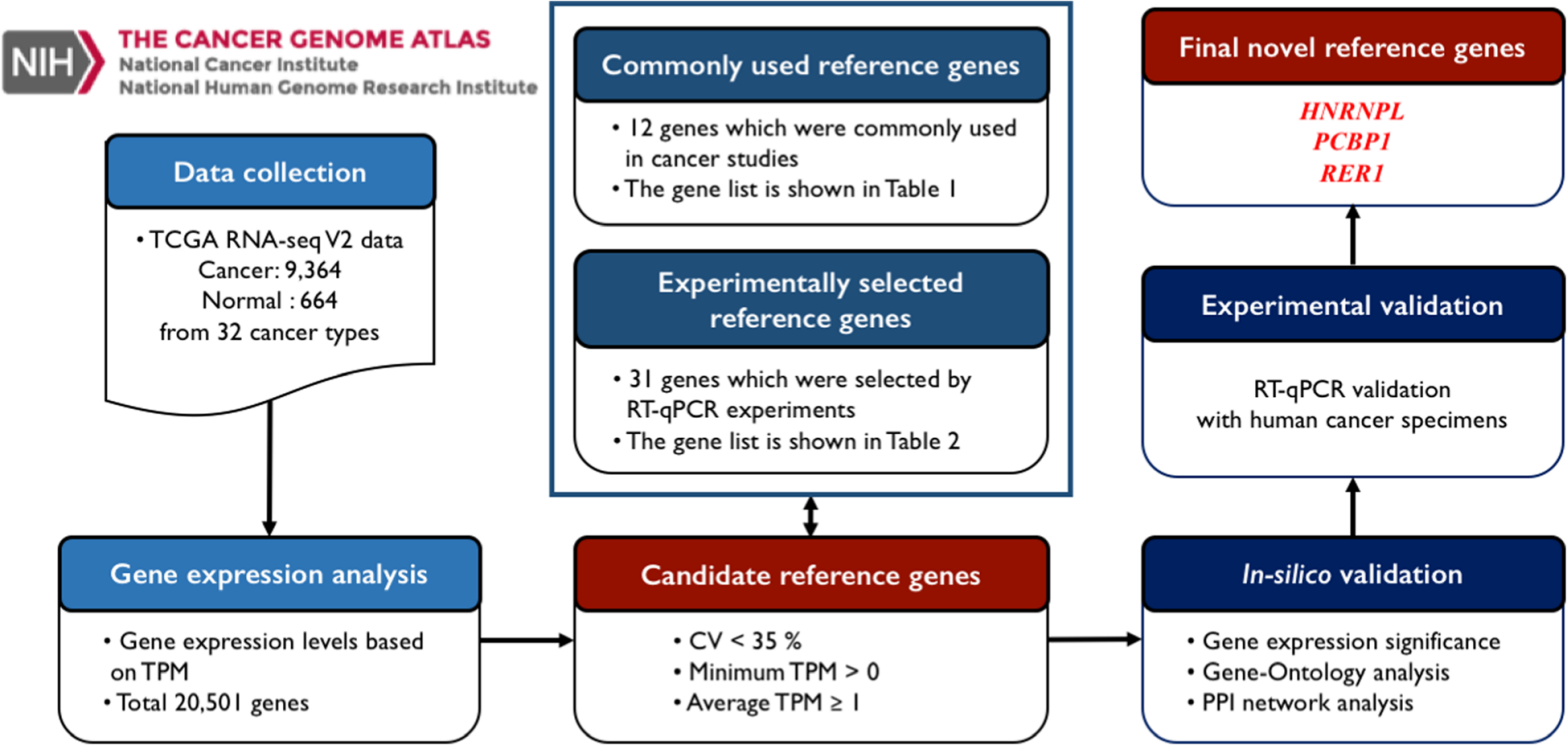
The overall workflow of the present study

The overall workflow of the present study is shown in Fig. 1. We downloaded RNA-sequence (RNA-seq) V2 data (level 3) of 34 different cancer types from the TCGA database (http://tcga-data.nci.nih.gov/tcga/). The TCGA RNA-seq pipeline has used two distinct measurement methods, comprising RPKM (*R*eads *P*er *K*ilobase per *M*illion mapped reads) [11] and TPM (*T*ranscripts *P*er *M*illion) [12, 13], to obtain expression levels from RNA-seq data. Given that TPM is established to produce more comparable results across various sample types than RPKM [13, 14], we used TPM-generated data for 32 of the 34 cancer types for further analyses [esophageal carcinoma (ESCA) and stomach adenocarcinoma (STAD) were excluded, since only RPKM-generated data were available for these cancer types]. Unless otherwise stated, all gene expression levels used in our analyses represent the unit of transformed (multiplied by 10^6^) normalized read counts (extracted from TCGA files with the extension “rsem.genes.normalized_results”).

The human protein interaction network data were collected from the Human Protein Reference Database (HPRD release 9, http://www.hprd.org) [15], which includes 30,047 protein entries and 41,327 protein-protein interactions (PPIs). We extracted all binary PPIs from the HPRD, and counted the number of interactions for each protein without redundancy to estimate the size of the protein complex.

We categorized the selected reference genes according to gene ontology groups using PANTHER (http://www.pantherdb.org/) [16] and DAVID (http://david.abcc.ncifcrf.gov/) [17] tools.

### Human specimens

The validity of all matched human cancerous and normal tissues was confirmed via patient clinical diagnosis. In total, 58 matched sample pairs were obtained for analysis, of which the cancerous tissue sample in each was isolated from patient breast (*n* = 18), colon (*n* = 12), thyroid (*n* = 8), lung (n = 8), liver (n = 8), kidney (*n* = 2), or cervical (n = 2) cancer tissues. All human tissue was trimmed to 0.5 cm^2^ immediately after removal from the patient and stored in 5 volumes of RNAlater solution (ThermoFisher Scientific, USA) at − 80 °C. For the experiment, samples were used within 3 years of storage. These all utilized human specimens and data were provided by the Biobank of Chungnam University Hospital (Korea Biobank Network).

### RNA preparation and RT-qPCR

Total RNA was extracted using a eCube Tissue RNA Mini Kit (PhileKorea, Korea) according to the manufacturer’s instructions, and reverse-transcribed using M-MLV reverse transcriptase (Promega, USA) with random hexamers. RT-qPCR was performed with a SYBR-Green fluorescent dye (GENET BIO, Korea) and the AriaMx PCR System (Agilent, USA). All reactions occurred under identical cycling conditions, comprising 40 cycles of amplification with denaturation (95 °C, 20 s), annealing (58 °C, 20 s), and elongation (72 °C, 20 s). The specificity of the products generated by each primer set was confirmed by both gel electrophoresis and a melting curve analysis (Additional file 1: Table S1 and Additional file 2: Figure S1).

## Results and discussion

### Commonly used reference genes exhibit a high level of expression variation in both tumorous and normal tissue samples

To assess the gene expression variability within human cancerous and normal tissues, we collected gene expression data from the TCGA database, which contains 10,028 (9,364 cancerous and 664 normal) samples isolated from 32 different cancer types. We used TPM-generated data to calculate the coefficient of variation (CV, calculated as the standard deviation divided by the mean), for target gene expression levels across the analyzed samples. We initially evaluated the gene expression variability of commonly used reference genes (Table 1) [18], and found all 12 analyzed genes to exhibit a CV-value greater than 45% (Table 1). Most (23/31, 74%; Tables 2 and 3) of the experimentally selected reference genes expressed in cancer tissues were observed to exhibit a similar level of gene expression variability. We repeated this process to separately analyze cancerous and normal samples, so as to eliminate potential error caused by sample size bias (since 9,364 cancerous, but only 664 normal tissue samples were analyzed). The results of this second analysis showed the same trends in each cancer and normal group, whereby all 12 commonly used reference genes and 74% (23/31) of the experimentally selected reference genes were found to exhibit a CV value greater than 45% in both groups together (Additional file 3: Table S2). These results suggest that the reference genes most commonly used in current cancer studies may not be appropriate to serve as representative reference genes, and thus, their use may lead to erroneous quantification of cancer-related gene expression levels.

**Table 1:** List of commonly used reference genes and their gene expression variability in 10,028 analyzed samples from TCGA database

Gene name	Description	Mean TPM value	CV(%)
ACTB	Actin Beta	4713.56	45.03
PGAM1	Phosphoglycerate Mutase 1	239.83	56.36
ALDOA	Aldolase, Fructose-Bisphosphate A	1576.45	57.97
TUBA1B	Tubulin Alpha 1b	974.69	62.63
HPRT1	Hypoxanthine Phosphoribosyltransferase 1	46.04	63.46
GAPDH	Glyceraldehyde-3-Phosphate Dehydrogenase	5445.97	67.55
B2M	Beta-2-Microglobulin	5083.41	69.57
PGK1	Phosphoglycerate Kinase 1	365.70	69.98
LDHA	Lactate Dehydrogenase A	575.04	79.40
PFKP	Phosphofructokinase, Platelet	94.20	102.74
VIM	Vimentin	1405.17	117.90
G6PD	Glucose-6-Phosphate Dehydrogenase	64.06	138.24

**Table 2:** List of experimentally selected reference genes

Tissues	Experimentally selected reference genes	References
Breast	PUM1, TBP, RPLP0, MRPL19, ACTB, SDHA, RPS23, HUWE1, EEF1A1, SF3A1, PPIA	[23–26]
Colon	B2M, PPIA, HPRT1, IPO8, HSP90AB1, YWHAZ, RPS13	[7, 27, 28]
Liver	HMBS, UBC, TBP, HPRT1, CTBP1	[29–33]
Lung	HPRT1, RPLP0, UBC, GAPDH, CASC3, PES1, POLR2A, YAP1, ACTB, EEF1A1, FAU, RPS9, RPS11, RPS14	[34–37]
Kidney	PPIA, RPS13, TBP	[38, 39]
Prostate	HPRT1, GAPDH, SDHA	[40, 41]
Thyroid	ACTB	[42]
HNS ^a^	GAPDH, RPS18, SDHA, ALAS1	[43]

^a^HNS: Head and Neck squamous cell

**Table 3:** Gene expression variability of experimentally selected reference genes in 10,028 TCGA database

Gene name	Mean TPM	STDEV	CV (%)
FAU	1392.44	538.16	38.65
CTBP1	114.52	44.26	38.65
RPS13	1571.26	675.69	43.00
UBC	1320.33	569.84	43.16
PUM1	35.09	15.17	43.22
RPS11	3969.12	1752.48	44.15
TBP	12.82	5.72	44.60
SF3A1	45.63	20.37	44.65
ACTB	4713.56	2122.56	45.03
RPS23	299.30	135.68	45.33
MRPL19	34.12	15.71	46.04
RPS14	2799.60	1316.81	47.04
IPO8	21.25	10.10	47.54
RPS9	2121.51	1065.24	50.21
PES1	66.70	34.82	52.20
RPLP0	1869.13	976.37	52.24
HUWE1	69.01	36.87	53.43
HMBS	28.07	15.41	54.91
POLR2A	60.70	33.65	55.43
HSP90AB1	1004.81	560.40	55.77
PPIA	187.35	108.62	57.98
EEF1A1	2661.35	1585.93	59.59
HPRT1	46.04	29.22	63.46
SDHA	103.74	66.11	63.72
YWHAZ	490.69	321.32	65.48
RPS18	5059.90	3336.16	65.93
GAPDH	5445.97	3678.69	67.55
B2M	5083.41	3536.78	69.57
CASC3	45.64	36.59	80.17
ALAS1	53.97	66.85	123.86
YAP1	44.89	66.00	147.04

### Selection of novel reference gene candidates from the TCGA database

**Fig. 2:**
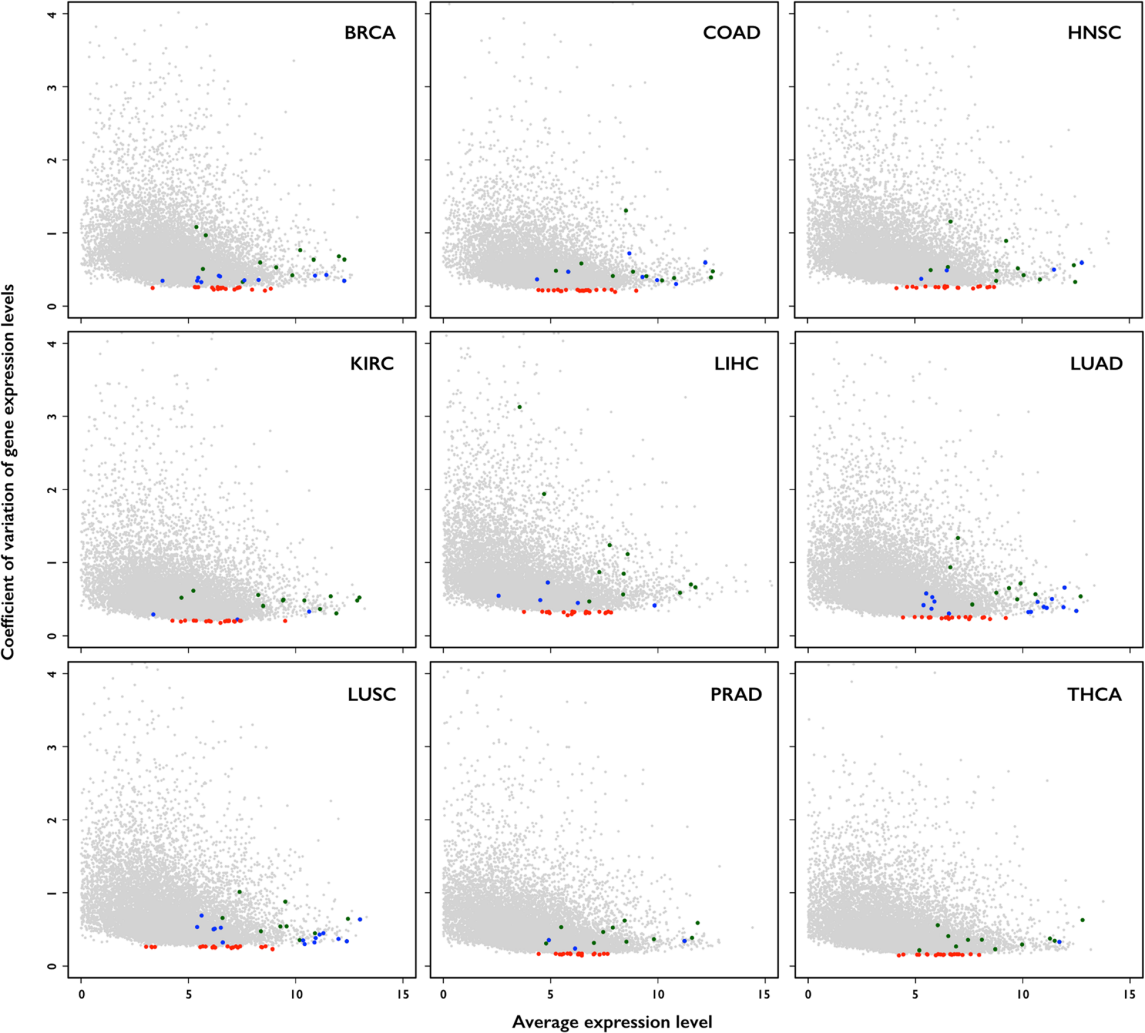
Distribution of coefficient of variation (CV) of gene expression levels in nine cancer data sets. Red color indicates top-ranked (by CV) 20 genes. Green and Blue colors indicate commonly used and experimentally selected reference genes (Tables 1 and 2), respectively

Because genetic alterations in diverse cancer types may differentially affect cellular processes at the transcriptome level, we investigated whether reference genes defined by analysis of a single type of cancerous tissue could be applied to other cancer types. Thus, we calculated and compared the CV values of > 40 samples (and their matched normal tissue samples) from nine cancer types (BRCA, COAD, HNSC, LUAD, LUSC, LIHC, PRAD, THCA, and KIRC; Additional file 4: Figure S2), that were contained within the TCGA database. Among a total set of 20 top-ranked (by CV) genes from each cancer type, no genes (1) were included in the list of commonly used reference genes, and (2) were found in more than 50% (5 out of 9) of cancer types (Fig. 2 and Additional file 5: Table S3), indicating the dependency of reference genes on cancer types.

**Fig. 3:**
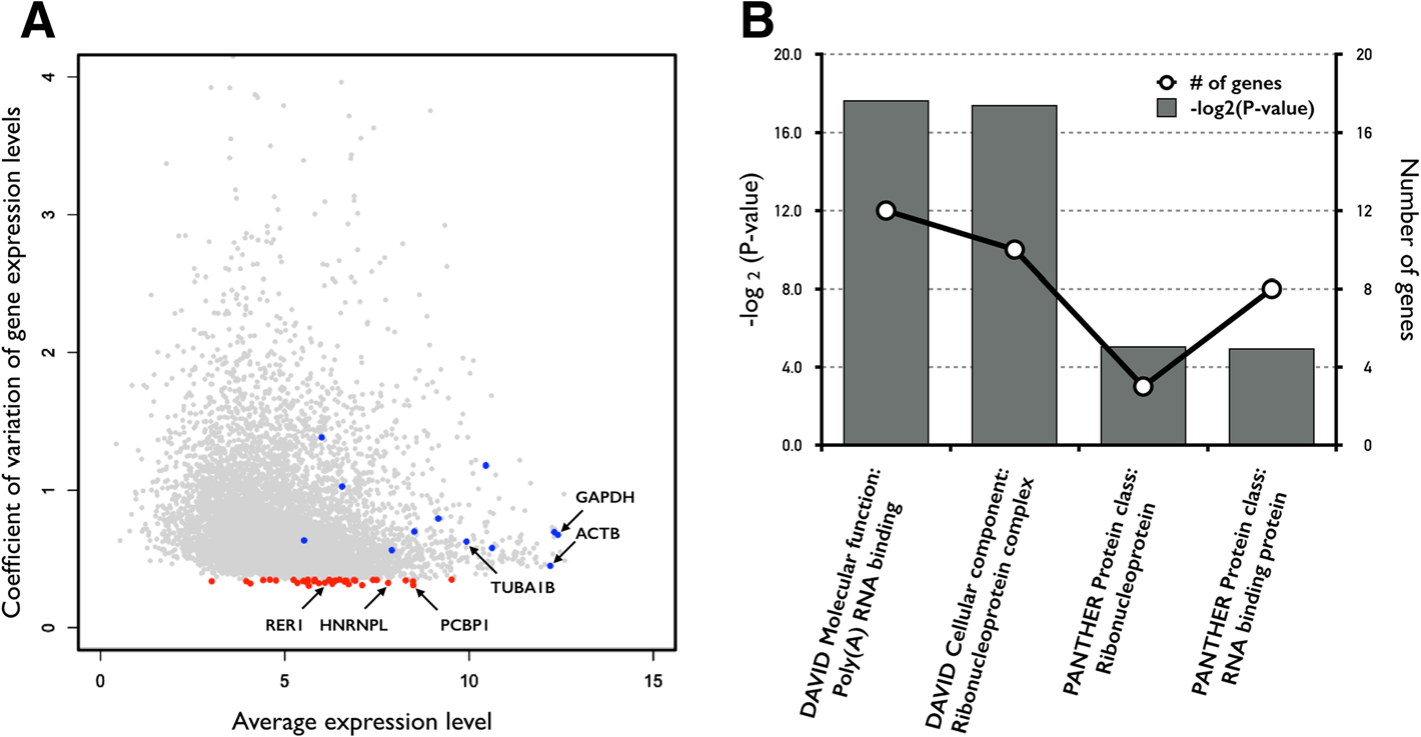
**a** Distribution of the coefficient of variation (CV) of gene expression levels in the analyzed cancerous and normal tissues. Red color indicates newly identified reference genes that have a CV value < 35%. Blue color indicates commonly used reference genes (Table 1). **b** Gene Ontology (GO) analysis of the newly identified reference genes

To newly determine suitable novel genes appropriate to act as internal controls for the normalization of target gene expression in cancer research, we selected a number of genes identified (1) to exhibit unvarying expression levels across both cancerous and normal tissue samples, (2) to have a CV value < 35%, (3) a minimum TPM > 0, (4) and an average of TPM value ≥1 across all tissue samples. Of the 10,028 analyzed samples from the 32 different cancer types, we identified 38 candidate novel cancer-research reference genes (Fig. 3a, Additional file 1: Table S4). We subsequently evaluated whether these newly identified reference genes had the same functional characteristics as the previously established, commonly used reference genes. We found the average expression level of the newly identified reference genes to be significantly higher than that of the others (115.06 versus 42.93; *P* < 0.0413, using an empirical permutation test with 10,000 replications). This result is consistent with previously reported expression levels for the established reference genes [4]. Next, we determined that, as expected [4, 5, 19], the newly identified reference genes were significantly enriched in functional categories associated with transcription-translation processes, such as polyA-RNA, ribonucleoprotein, and RNA-binding (FDR < 5%, Fig. 3b). The established reference genes have been previously demonstrated to act as the ‘hubs’ of the highly connected protein-protein interaction (PPI) networks [20–22]. In the present study, we observed the newly identified reference genes to be characterized by a greater number of PPI network-interaction partners than the other genes (8.42 versus 3.67; *P* < 0.0185, using an empirical permutation test with 10,000 replications), indicating their functional importance for biological systems.

### RT-qPCR validation of the newly identified reference genes in human cancer tissues

We next sought to confirm the validity of the newly identified candidates as reference genes for the normalization of RT-qPCR expression data in the context of human cancer. Therefore, we compared the RT-qPCR analysis results for two commonly used reference genes (*GAPDH* and *β-actin*) with those for the 11 most highly expressed of the newly identified reference genes (*PCBP1, HNRNPC, HNRNPL, EMC4, SNX17, MRPL43, IST1, FAM32A, PFDN1, RNF10,* and *RER1*) across 29 patient samples including breast, colon, liver, lung, and/or thyroid cancer types. Each human tissue was immersed in RNAlater solution immediately after extraction from the patient and stored at -80 °C to minimize RNA degradation. In addition, 2 μg of total RNA extracted from tissues was electrophoresed on 1.5% denaturing agarose gel and only 28S/18S ratio of > 2 confirmed RNA was used in the experiment. The specificity of the products generated by each primer set was confirmed by both gel electrophoresis and a melting curve analysis (Additional file 1: Table S1 and Additional file 2: Figure S1).

**Fig. 4:**
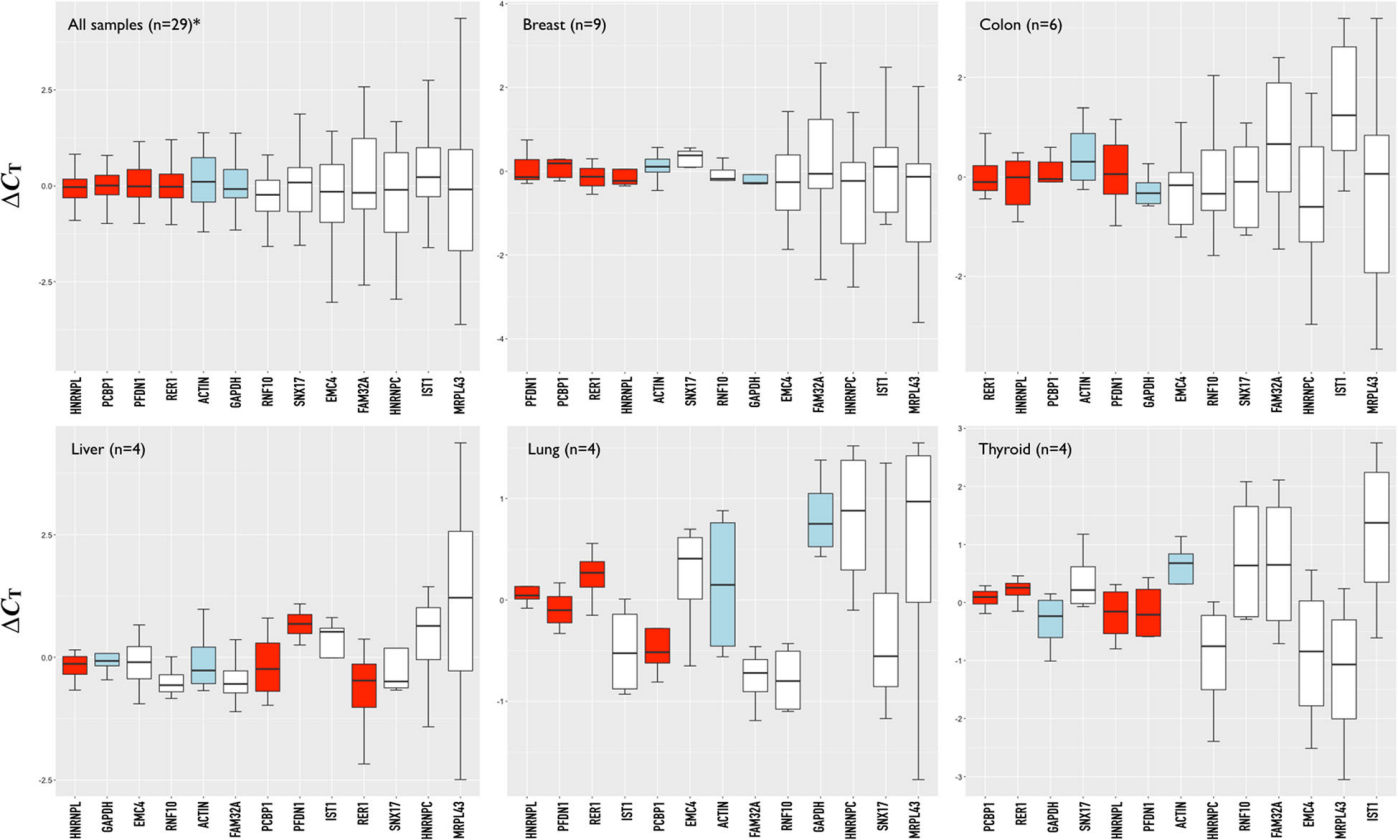
Validation of the gene expression variability of the novel reference genes by RT-qPCR. RT-qPCR analyses for two commonly used reference genes (*GAPDH* and *β-actin*, light blue-colored box), and 11 newly identified reference genes (*PCBP1, HNRNPC, HNRNPL, EMC4, SNX17, MRPL43, IST1, FAM32A, PFDN1, RNF10,* and *RER1*) that were highly ranked among the 38 analyzed genes according to their expression levels (indicated by their calculated CV values). *ΔC*_T_ indicates average difference of *C*_T_ value between cancerous and normal tissue samples (i.e., *C*_T [cancer]_ - *C*_T [normal]_). Newly identified reference genes whose *ΔC*_T_ value was found to be lower than that of both *β-actin* and *GAPDH* in all samples are highlighted (red-colored box). ^*^Samples from seven types of cancerous tissues, including breast (*n* = 9), colon (*n* = 6), liver (*n* = 4), lung (n = 4), thyroid (n = 4), kidney (*n* = 1), and cervical (n = 1) were combined. Note that kidney and cervical tissues have not been separately represented in the box plot

Since optimal references genes for cancer-transcriptome analysis should exhibit a low level of expression variability between cancerous and normal tissue samples, we isolated total RNA from each cancerous and normal sample from a single patient and compared their *C*_T_ values (where, *C*_T_ is the “*C*ycle Threshold”, defined as the number of cycles required for the fluorescence signal to exceed background level, and is inversely correlated with the amount of target nucleic acid in the sample). Of the 11 newly identified genes, *HNRNPL* (*ΔC*_T_ = 0.37), *PCBP1* (*ΔC*_T_ = 0.42), *PFDN1* (*ΔC*_T_ = 0.46), and *RER1* (*ΔC*_T_ = 0.48) were found to have a lower average *C*_T_ difference (*ΔC*_T_ = *C*_T [cancer]_ - *C*_T [normal]_) between cancerous and normal tissue samples than *β-actin* (*ΔC*_T_ = 0.58) and/or *GAPDH* (*ΔC*_T_ = 0.60), suggesting their suitability for use as consensus reference genes for gene expression studies in human cancer (Fig. 4). To ensure the reliability and robustness of these results, we reconfirmed whether these reference genes had lower *ΔC*_T_ values than *β-actin* and/or *GAPDH* in each cancer sample. *HNRNPL* was identified to have a *ΔC*_T_ value lower than that of both *β-actin* and *GAPDH* in four (breast, colon, liver, and lung) of five cancer sample types. Similarly, *PCBP1* and *RER1* had lower *ΔC*_T_ values than *β-actin* and *GAPDH* in all cancer sample types except liver cancer tissue, and *PFDN1* exhibited a lower *ΔC*_T_ value than *β-actin* and *GAPDH* in two cancer sample types (breast and lung, Fig. 4).

## Conclusion

In summary, cancer is a disease characterized by complex molecular networks, in which highly heterogeneous and multifocal tumor cells cooperate with host cells within their microenvironment. Recent gene expression studies have been conducted to investigate the intricate interplay of gene expression patterns that regulate cancer invasion and metastasis at the transcriptional level; however, their accurate quantification of gene expression level is dependent upon the selection and use of reliable and appropriate reference genes for the normalization of target gene expression levels. Thus, in the present study, we performed in silico bioinformatics analyses and experimental validation to identify *HNRNPL*, *PCBP1* and *RER1* as novel candidate reference genes, whose expression is predominantly consistent, independent of cancer type, stage, and treatment status, and of patient age and gender. Although a larger sample size and more cancer types are needed for more reliable results, these novel reference genes will be invaluable for diagnosis and the prediction of patient prognosis, in a wide range of human cancers.

## Additional files

Additional file 1: **Table S1.** Primers used for quantitative analysis of gene expression. **Table S4.** Gene expression variability of newly identified reference genes. (DOCX 28 kb)

Additional file 2: **Figure S1.** qPCR electrophoresis result and melting curve analysis of our reference genes. (A) Agarose gel electrophoresis showing specific reverse transcription PCR products of the expected size for each gene. (B) Melting curves generated for all genes. (TIFF 34570 kb)

Additional file 3: **Table S2.** Gene expression variability of commonly used and experimentally selected reference genes in each cancerous and normal group. (XLSX 24 kb)

Additional file 4: **Figure S2.** Nine cancer types. Nine cancer types from TCGA comprising both cancerous and matched normal data with > 40 samples. (TIFF 3075 kb)

Additional file 5: **Table S3.** Top 20 candidate reference genes in each cancer type. (XLSX 65 kb)

## Abbreviations

BRCA: Breast invasive carcinoma
COAD: Colon adenocarcinoma
CV: Coefficient of variation
FDR: False discovery rate
HNSC: Head and neck squamous cell carcinoma
IRB: Institutional review board
KIRC: Kidney renal clear cell carcinoma
LIHC: Liver hepatocellular carcinoma
LUAD: Lung adenocarcinoma
LUSC: Lung squamous cell carcinoma
M-MLV: Moloney murine leukemia virus
PRAD: Prostate adenocarcinoma
RPKM: Reads per kilobase per million mapped reads
RT-qPCR: Quantitative reverse transcription PCR
TCGA: The cancer genome atlas
THCA: Thyloid carcinoma
TPM: Transcripts per million
