# Supplementary material for: Conventionally used reference genes are not outstanding for normalization of gene expression in human cancer research
Source: BMC Bioinformatics. 2019 May 29;20(Suppl 10):245. doi: 10.1186/s12859-019-2809-2 (PMC6538551; doi:10.1186/s12859-019-2809-2)
Supplement: Supplementary file 1 — Table S1. Primers used for quantitative analysis of gene expression. Table S4. Gene expression variability of newly identified reference genes. (DOCX 28 kb) [file 12859_2019_2809_MOESM1_ESM.docx]

**Supplementary Tables**

**Conventionally used reference genes are not outstanding for normalization of gene expression in human cancer research**

Jihoon Jo, Sunkyung Choi, Jooseong Oh, Sung-Gwon Lee, Song Yi Choi, Kee K. Kim, and Chungoo Park

Supplementary Tables S1 and S4

**Supplementary Table S1. Primers used for quantitative analysis of gene expression**

| **Gene** |  | **Primer sequence (5’ to 3’)** | **Product size (bp)** | **Annealing temp. (℃)** |
| --- | --- | --- | --- | --- |
| **PCBP1** | Forward | TGA TCA TCG ACA AGC TGG AG | 145 | 58 |
|  | Reverse | TCT TTG ATC TTA CAC CCG CC |  |  |
| **HNRNPC** | Forward | TGC AGT GGA AGG AAA CAC AA | 142 | 58 |
|  | Reverse | GAG GAG GAG CAG AGA ATG GA |  |  |
| **HNRNPL** | Forward | ACA AAC CCC AAT CTC AGT GG | 140 | 58 |
|  | Reverse | CCC TCA TCA TGG TAA TGG CT |  |  |
| **EMC4** | Forward | TGA TGG GTT TGG CAT TGG CT | 129 | 58 |
|  | Reverse | ACA AAA GCA GTC CTC CAC CA |  |  |
| **SNX17** | Forward | CGC CTA CGT GGC CTA TAA CA | 144 | 58 |
|  | Reverse | GAG AAA AGC TTC TTT GGG GG |  |  |
| **MRPL43** | Forward | GGA CAC TGG CCT GAG ACT GT | 150 | 58 |
|  | Reverse | ATC GGG AGG CAA GAA ACT G |  |  |
| **IST1** | Forward | CAC GGC AGG ATT CGG TTA | 133 | 58 |
|  | Reverse | GGC CAG TTC CGT TTT CTT TT |  |  |
| **FAM32A** | Forward | CCT TCG AGA AAA TGC AGG AG | 137 | 58 |
|  | Reverse | GCT GAC TTT GGG AAT GTC GT |  |  |
| **PFDN1** | Forward | TCC AAG GAA GCA ATT CAC AG | 146 | 58 |
|  | Reverse | CTT CGT GCC ATC AGC ATC T |  |  |
| **RNF10** | Forward | CGA GTC TAA ACC CAA GAG CG | 134 | 58 |
|  | Reverse | TCT TGC TTT TCT GTG AAC TGG A |  |  |
| **RER1** | Forward | CGT AGC GGA GCT GCG AG | 144 | 58 |
|  | Reverse | CGT GTA GGG TGT GGA CTT GT |  |  |
| **GAPDH** | Forward | TTG AGG TCA ATG AAG GGG TC | 117 | 58 |
|  | Reverse | GAA GGT GAA GGT CGG AGT CA |  |  |
| **β-actin** | Forward | TCA CCC ACA CTG TGC CCA TCT ACG A | 295 | 58 |
|  | Reverse | CAG CGG AAC CGC TCA TTG CCA ATG G |  |  |

**Supplementary Table S4. Gene expression variability of newly identified reference genes**

| **Gene name** | **Mean** | **Max** | **Min** | **STDEV** | **CV (%)** |
| --- | --- | --- | --- | --- | --- |
| **SDHAF2** | 50.2491 | 171.5242 | 15.3085 | 4.5074 | **0.3047** |
| **EMC4** | 137.2030 | 500.3495 | 42.3423 | 16.5118 | **0.3086** |
| **PCBP1** | 358.6714 | 1186.7919 | 110.8220 | 20.8802 | **0.3090** |
| **SNX17** | 106.4122 | 330.7849 | 33.6434 | 9.976 | **0.3162** |
| **RNF10** | 78.2617 | 268.5496 | 24.9716 | 10.3162 | **0.3191** |
| **C10orf76** | 16.7689 | 70.6062 | 5.3823 | 2.4592 | **0.3210** |
| **TARDBP** | 60.8616 | 178.1058 | 19.6700 | 4.2863 | **0.3232** |
| **MRPS18C** | 40.5862 | 143.6075 | 13.1810 | 4.2365 | **0.3248** |
| **HNRNPL** | 223.4807 | 885.2372 | 72.6633 | 26.2495 | **0.3251** |
| **RER1** | 68.2847 | 582.6490 | 22.2861 | 11.8422 | **0.3264** |
| **TMEM203** | 47.2489 | 190.6764 | 15.8081 | 5.6501 | **0.3346** |
| **FAM32A** | 98.4605 | 696.8181 | 32.9555 | 14.9345 | **0.3347** |
| **HNRNPC** | 356.2767 | 939.0961 | 119.6989 | 34.3837 | **0.3360** |
| **HARS** | 55.5641 | 423.0781 | 18.7384 | 5.4913 | **0.3372** |
| **NAIF1** | 8.0905 | 33.1526 | 2.7305 | 0.0906 | **0.3375** |
| **IST1** | 98.6429 | 502.1788 | 33.2976 | 10.4036 | **0.3376** |
| **RRP8** | 15.5211 | 55.7801 | 5.2439 | 0.9347 | **0.3379** |
| **HDAC3** | 45.3154 | 155.9307 | 15.3605 | 3.5504 | **0.3390** |
| **MRPL43** | 102.3956 | 423.7694 | 34.8115 | 12.9299 | **0.3400** |
| **PFDN1** | 82.2310 | 540.8775 | 28.0299 | 9.1064 | **0.3409** |
| **C19orf50** | 82.5215 | 445.7121 | 11.0340 | 28.1902 | **0.3416** |
| **SPG21** | 100.5787 | 573.7196 | 12.7235 | 34.3982 | **0.3420** |
| **MRPS5** | 49.2242 | 310.4236 | 3.2800 | 16.8398 | **0.3421** |
| **RNF167** | 120.5790 | 547.5574 | 8.7893 | 41.2521 | **0.3421** |
| **DNAJB12** | 27.1179 | 223.0132 | 3.4704 | 9.3036 | **0.3431** |
| **HNRNPK** | 310.5811 | 1215.0154 | 27.2345 | 106.6527 | **0.3434** |
| **PSMF1** | 74.7089 | 374.2952 | 6.2864 | 25.7122 | **0.3442** |
| **C1orf212** | 21.3357 | 89.3778 | 2.7083 | 7.3724 | **0.3455** |
| **AAMP** | 117.5885 | 431.5582 | 5.7112 | 40.701 | **0.3461** |
| **UBE2D3** | 180.6354 | 845.7154 | 18.4130 | 62.5477 | **0.3463** |
| **EIF4H** | 168.6820 | 711.7380 | 7.2054 | 58.4987 | **0.3468** |
| **CIAO1** | 37.8328 | 140.2641 | 5.0833 | 13.1319 | **0.3471** |
| **DNAJC7** | 73.8051 | 441.1302 | 6.2104 | 25.655 | **0.3476** |
| **STX5** | 49.3510 | 206.0820 | 3.0621 | 17.1983 | **0.3485** |
| **ELAVL1** | 24.1675 | 87.3564 | 3.0930 | 8.4275 | **0.3487** |
| **BCL7B** | 56.2344 | 246.9183 | 5.8724 | 19.6243 | **0.3490** |
| **DNAJC8** | 89.6514 | 307.0350 | 9.5486 | 31.3139 | **0.3493** |
| **EIF1** | 737.2460 | 3741.9459 | 60.4643 | 257.7606 | **0.3496** |
