# Supplementary figures and images for: Conventionally used reference genes are not outstanding for normalization of gene expression in human cancer research
Source: BMC Bioinformatics. 2019 May 29;20(Suppl 10):245. doi: 10.1186/s12859-019-2809-2 (PMC6538551; doi:10.1186/s12859-019-2809-2)

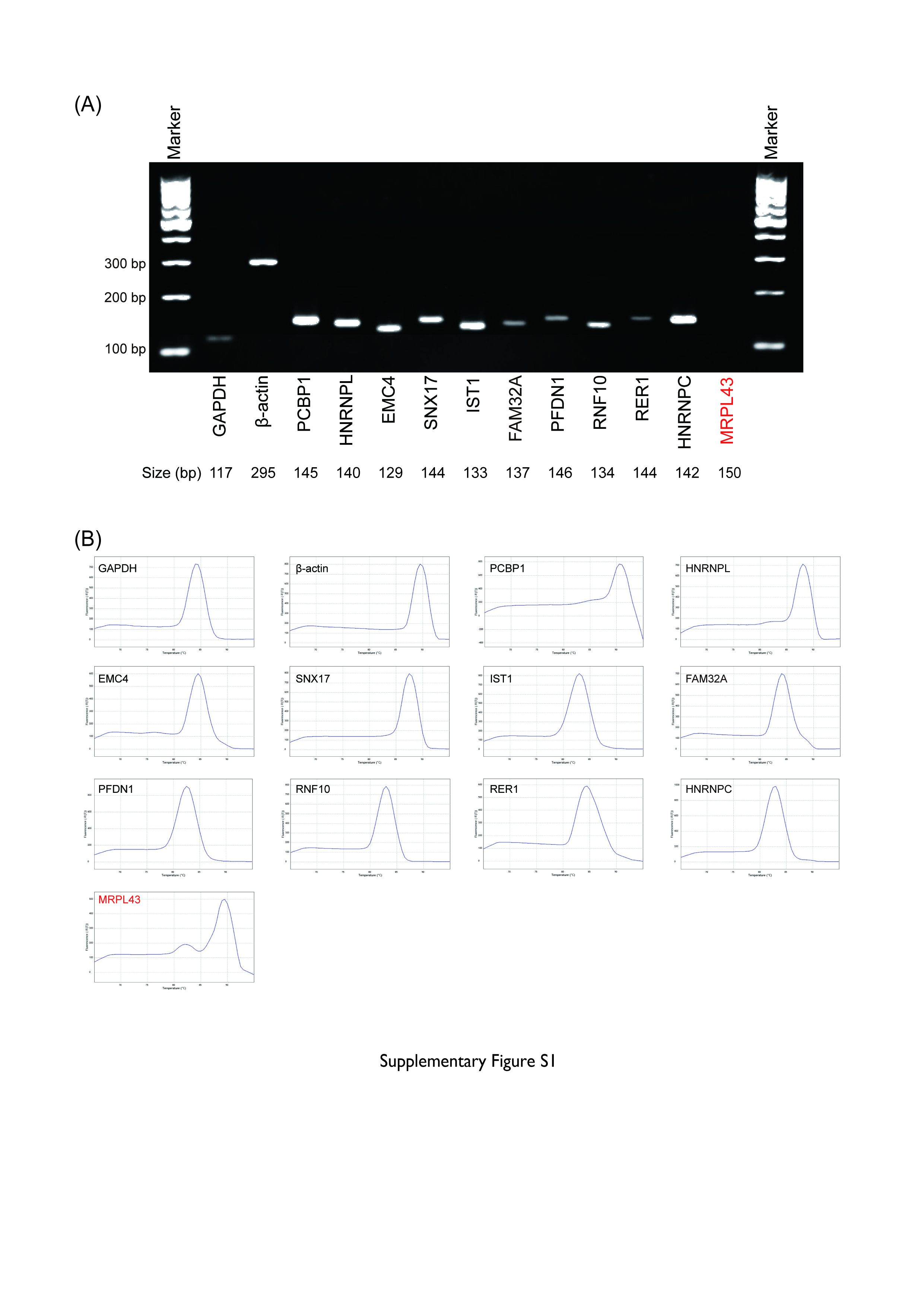

Supplement: Supplementary file 2 — Figure S1. qPCR electrophoresis result and melting curve analysis of our reference genes. (A) Agarose gel electrophoresis showing specific reverse transcription PCR products of the expected size for each gene. (B) Melting curves generated for all genes. (TIFF 34570 kb) [file 12859_2019_2809_MOESM2_ESM.tiff]

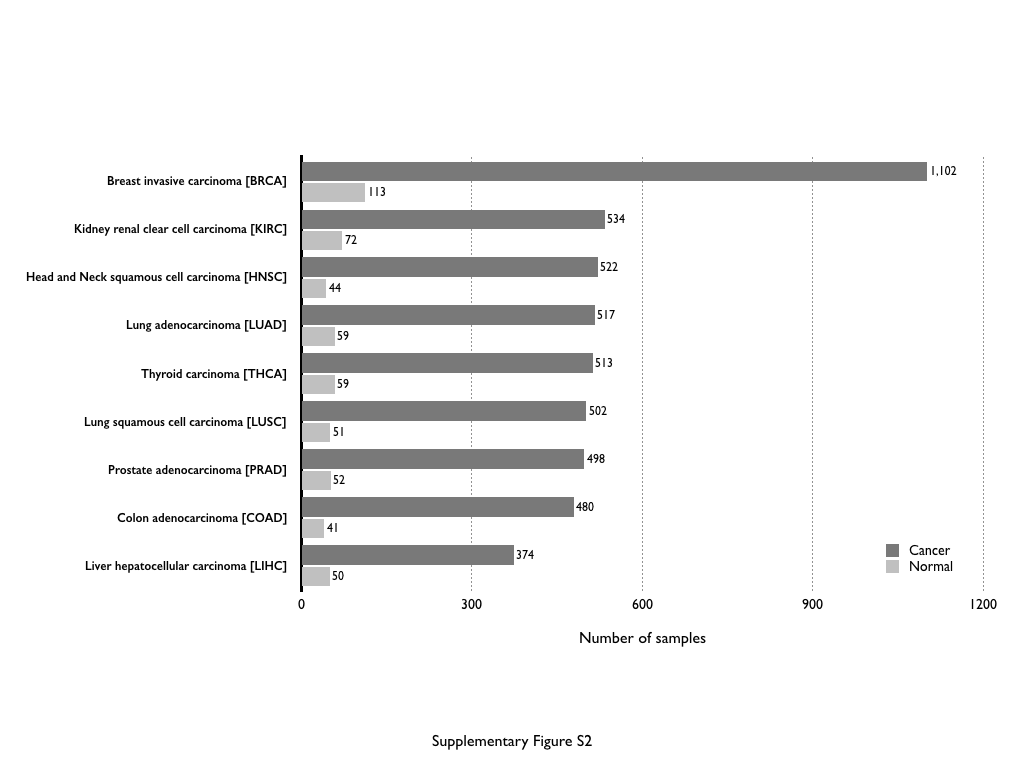

Supplement: Supplementary file 4 — Figure S2. Nine cancer types. Nine cancer types from TCGA comprising both cancerous and matched normal data with > 40 samples. (TIFF 3075 kb) [file 12859_2019_2809_MOESM4_ESM.tiff]
